# Supplementary material for: Development of test bench to determine the distribution of granular fertilizers in planting rows using spiral roller, two spiral rollers and fluted roller
Source: PLoS One. 2020 Dec 14;15(12):e0243799. doi: 10.1371/journal.pone.0243799 (PMC7735587; doi:10.1371/journal.pone.0243799)

Control chart layout model for speeds 1.11 m s^{-1}

	GF_1	GF_2
Spiral Single	Fig 4a	Fig 4b
Spiral Double	Fig 4c	Fig 4d
Fluted Roller	Fig 4e	Fig 4f

Control chart layout model for speeds 1.94 m s^{-1}

	GF_1	GF_2
Spiral Single	Fig 5a	Fig 5b
Spiral Double	Fig 5c	Fig 5d
Fluted Roller	Fig 5e	Fig 5f

Control chart layout model for speeds 2.77 m s^{-1}

	GF_1	GF_2
Spiral Single	Fig 6a	Fig 6b
Spiral Double	Fig 6c	Fig 6d
Fluted Roller	Fig 6e	Fig 6f

Example speed 1.11 m s^{-1}

GF$_1$ | GF$_2$

Spiral Single

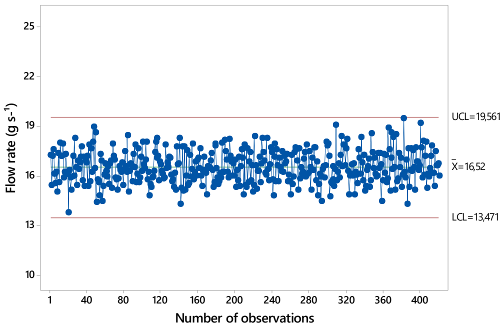

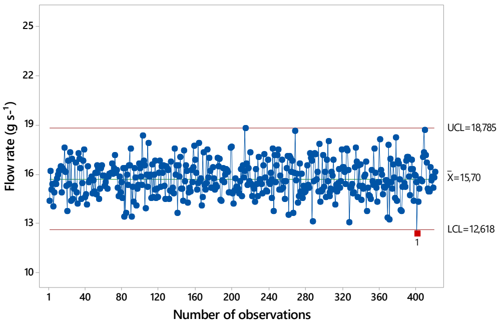

Spiral Double

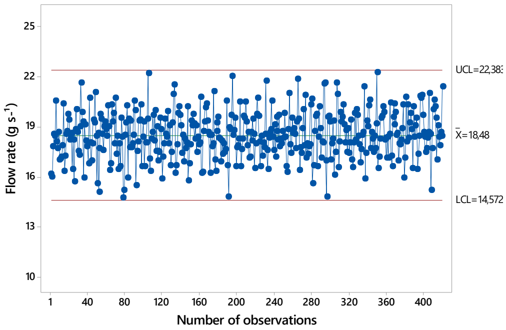

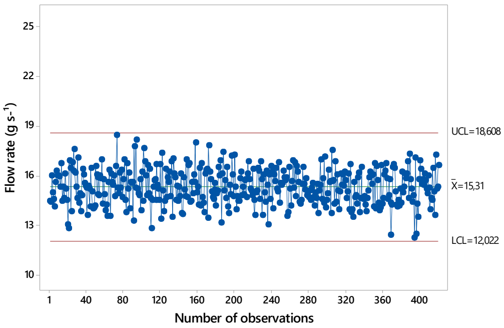

Fluted Roller

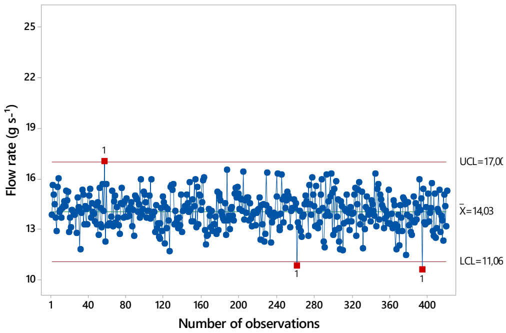

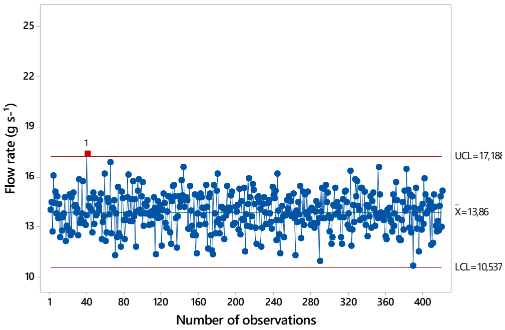

Supplement: S1 Raw images — (PDF) [file pone.0243799.s001.pdf]
