# Supplementary material for: Development of test bench to determine the distribution of granular fertilizers in planting rows using spiral roller, two spiral rollers and fluted roller
Source: PLoS One. 2020 Dec 14;15(12):e0243799. doi: 10.1371/journal.pone.0243799 (PMC7735587; doi:10.1371/journal.pone.0243799)
Supplement: Attachment — Submitted filename: Response to Reviewers.pdf [file pone.0243799.s002.pdf]

Response to Reviewers

Review Comments to the Author

Reviewer #1: The title of the article does not correspond to the content. The article lacks data on the cost of the test bench and on the cost of the study.

In the presented figures 4a-4f and 5a-5f, it is necessary to expand the distribution of data along the Y-axis. This is necessary for greater clarity of the figures.

In the conclusions and annotations, it is necessary to indicate the cost of the test bench and how cheap it is to work in comparison with analogs.

The title of the manuscript was changed, removing the term low cost, and the economic direction of the development of the structure and its quotations. A new paragraph was added in the introduction, referring to the justification and importance of the work. The expansion of the data distribution of the commented figures, could not be attended for statistical and software reasons. As for the cost results in the conclusion, it will no longer be necessary due to the change in the title of the manuscript.

Reviewer #2: The study sought to develop a low-cost test bench to determine the distribution of granular fertilizers in planting rows using spiral roller, two spiral rollers and fluted roller. The introductory section of the manuscript appears disjointed and incoherent. The aim of the study is conspicuously not made clear in the write-up. The authors failed to compare the output flow rates of the developed test bench with already existing spreaders. What was the basis for comparison relative to the efficiency of this newly developed technology with already existing technologies? The authors again failed to support their findings with what has been done elsewhere. The manuscript actually lacks the required literature review.

R: The title of the manuscript was changed, removing the term low cost, and the economic direction of the development of the structure and its quotations. A new paragraph was added in the introduction, referring to the justification and importance of the work. The output flow rates were addressed before the descriptive statistics of the data and were not compared to other literature because there were no methods of evaluating the spreaders yet, so it was not possible to add new changes. This test bench was developed for this proposal to evaluate the regularity of spreader distribution, and the existing works do not meet the technical conditions for comparison.
