# Supplementary material for: Development of test bench to determine the distribution of granular fertilizers in planting rows using spiral roller, two spiral rollers and fluted roller
Source: PLoS One. 2020 Dec 14;15(12):e0243799. doi: 10.1371/journal.pone.0243799 (PMC7735587; doi:10.1371/journal.pone.0243799)
Supplement: Attachment — Submitted filename: General comments_PONE-D-20-09797R1.docx [file pone.0243799.s003.docx]

**General comments**

This manuscript is an improved version of what was earlier on submitted. The comments raised by the reviewers in the previous review have been adequately addressed.

The development of test bench to determine the distribution of granular fertilizers will undoubtedly serve as a prototype that will improve the application and distribution of granular-based fertilizers for that will increase fertilizer use efficiency for enhanced ecosystem functions and services.
